# The impact of maternal intrahepatic cholestasis during pregnancy on the growth trajectory of offspring: a population-based nested case‒control cohort study

**DOI:** 10.1186/s12884-024-06559-z

**Published:** 2024-06-07

**Authors:** Xueqi Li, Yao Kong, Yuxin Ren, Yaqian Li, Jinfeng Xu, Yongchi Zhan, Shu Zhou, Fan Yang, Tingting Xu, Xiaodong Wang

**Affiliations:** 1 Department of Obstetrics and Gynecology, West China Second University Hospital, Sichuan University, Renmin Nan Road, Chengdu, 610041 Sichuan China; 2 Key Laboratory of Birth Defects and Related Diseases of Women and Children (Sichuan University), Ministry of Education, Chengdu, 610041 Sichuan China; 3 Department of Child Health, West China Second University Hospital, Sichuan University, Chengdu, China

**Keywords:** Intrahepatic cholestasis of pregnancy, Growth disparities, Catch-up growth, Child health

## Abstract

**Background:**

Intrahepatic cholestasis of pregnancy (ICP) is associated with an increased risk of adverse fetal outcomes, yet its influence on offspring growth remains unclear. Our study dynamically tracks growth rates in children from ICP and healthy mothers and investigates the link between maternal liver function and developmental abnormalities in offspring.

**Method:**

Our case‒control study involved 97 women with ICP and 152 with uncomplicated pregnancies nested in a cohort of their offspring, including 50 from the ICP group and 87 from the uncomplicated pregnancy group. We collected pediatric growth and development data, with a maximum follow-up duration of 36 months. Stratified analyses of children's height, weight, and head circumference were conducted, and Spearman's rank correlation was applied to examine the relationships between maternal serological markers and pediatric growth metrics.

**Result:**

Maternal liver and renal functions, along with serum lipid profiles, significantly differed between the ICP and normal groups. In the ICP group, the offspring showed elevated alanine aminotransferase (ALT), direct bilirubin (DBIT), high-density lipoprotein cholesterol (HDL-C), low-density lipoprotein cholesterol (LDL-C), and apolipoprotein B (APOB) levels. Notably, the length-for-age z score (LAZ), weight-for-age z score (WAZ), and head circumference-for-age z score (HCZ) were lower in ICP offspring compared with those from normal pregnancies within the 1- to 12-month age range (*P* < 0.05). However, no significant differences in LAZ, weight-for-length z score (WLZ), BMI-for-age z score (BAZ), or HCZ were observed between groups in the 13- to 36-month age range. Maternal maximum lactate dehydrogenase (LDH) and total bile acids (TBA) levels during pregnancy were inversely correlated with LAZ and WAZ in the first year. Furthermore, offspring of mothers with ICP exhibited a greater incidence of stunting (24% vs. 6.9%, *P* = 0.004) and abnormal HCZ (14% vs. 3.7%, *P* = 0.034).

**Conclusions:**

Growth disparities in offspring of ICP-affected pregnancies were most significant within the 1- to 12-month age range. During this period, maximum maternal LDH and TBA levels were negatively correlated with LAZ and WAZ values of offspring. The observation of similar growth rates between ICP and control group offspring from 13 to 36 months suggested catch-up growth in the ICP group.

**Supplementary Information:**

The online version contains supplementary material available at 10.1186/s12884-024-06559-z.

## Introduction

Intrahepatic cholestasis of pregnancy (ICP), also called “obstetric cholestasis”, is a multifactorial condition of pregnancy that occurs in the second or third trimester and is characterized by elevated serum bile acid and/or liver aminotransferase levels and pruritus in the absence of a skin rash [1]. The incidence of ICP varies based on ethnic group and geographical region. The risk factors for ICP include multiple pregnancies, advanced maternal age, in vitro fertilization-embryo transfer (IVF-ET), a history of prior pregnancy, and a family history of ICP. The exact etiology of ICP is not completely understood, but reproductive hormones and genetic, endocrine and environmental factors may account for its incidence [2, 3].

Adverse fetal outcomes include preterm birth, preterm rupture of membranes, meconium-stained amniotic fluid and even sudden stillbirth, which are significant concerns for both patients and clinicians [4, 5] Iatrogenic premature birth significantly elevates the incidence of premature birth in cases of ICP to prevent sudden stillborn infants. Higher levels of serum total bile acids (TBA) also contribute significantly to spontaneous premature birth [6].

Nevertheless, studies of ICP outcomes should not be limited to its short-term effects, exploring the long-term impacts of ICP on both mothers and their offspring is also crucial for advancing maternal–fetal medicine. Improving pregnancy health significantly benefits both maternal and offspring well-being. Maternal characteristics during pregnancy have a profound impact on fetal and child outcomes. The placenta plays an indispensable role in fetal development and can act as a selective barrier, critically regulating the transfer of maternal bile acids to the fetus, thereby preventing potential adverse effects induced by excessive exposure to these compounds [7]. Elevated bile acid may cause vasoconstriction and affect the development of the placental villous tree and its consequences for fetal growth [8, 9].

This connection between maternal health and subsequent child development underscores the importance of identifying and understanding these relationships [10]. One study revealed that ICP is typically not associated with severe maternal morbidity but may increase the risk of subsequent hepatobiliary disease [11]. Emerging studies have focused on the association between ICP patients and their offspring. Recent studies have shown that the ICP may increase the risk of abnormal neurodevelopment and heart function in children [12–15].

Although an association between ICP and a heightened risk of adverse obstetric and neonatal outcomes has been established, with evidence suggesting that higher serum TBA levels amplify this risk [6, 16], there appears to be a gap in the literature regarding the long-term impact of maternal ICP on the developmental trajectory of offspring. Moreover, disruption of bile acid homeostasis can affect metabolic regulatory mechanisms, potentially influencing lipid metabolism, insulin resistance, and renal function [17–21], but the effect of ICP on offspring metabolism has not been reported to date.

Hence, in this study, we aimed to assess the impact of ICP on offspring development, including growth, weight, and head circumference, by dynamically tracking pediatric data from the Department of Child Health, West China Second Hospital, Sichuan University. Additionally, we collected and analyzed biochemical indicators from both offspring and maternal blood serum to explore the potential metabolic effects of ICP, thereby highlighting the potential impact of ICP on child development and metabolic health.

## Methods

### Sample size calculation

This sample size calculation was conducted using G*Power software. Based on the set parameters of a medium effect size (Cohen's d = 0.5), a significance level (α = 0.05), and a statistical power of 80%, approximately 64 participants per group are needed. Thus, a total of approximately 128 participants are required to ensure that the study has sufficient statistical power to detect the anticipated effect size, assuming a two-sided test design. Considering potential losses due to follow-up, we decided to double the initial calculated sample size to enhance the robustness of our study outcomes. Consequently, with a case to control ratio of approximately 1:1.5, the final sample size will include 97 participants with ICP and 152 control participants.

### Maternal study population

**Fig. 1:**
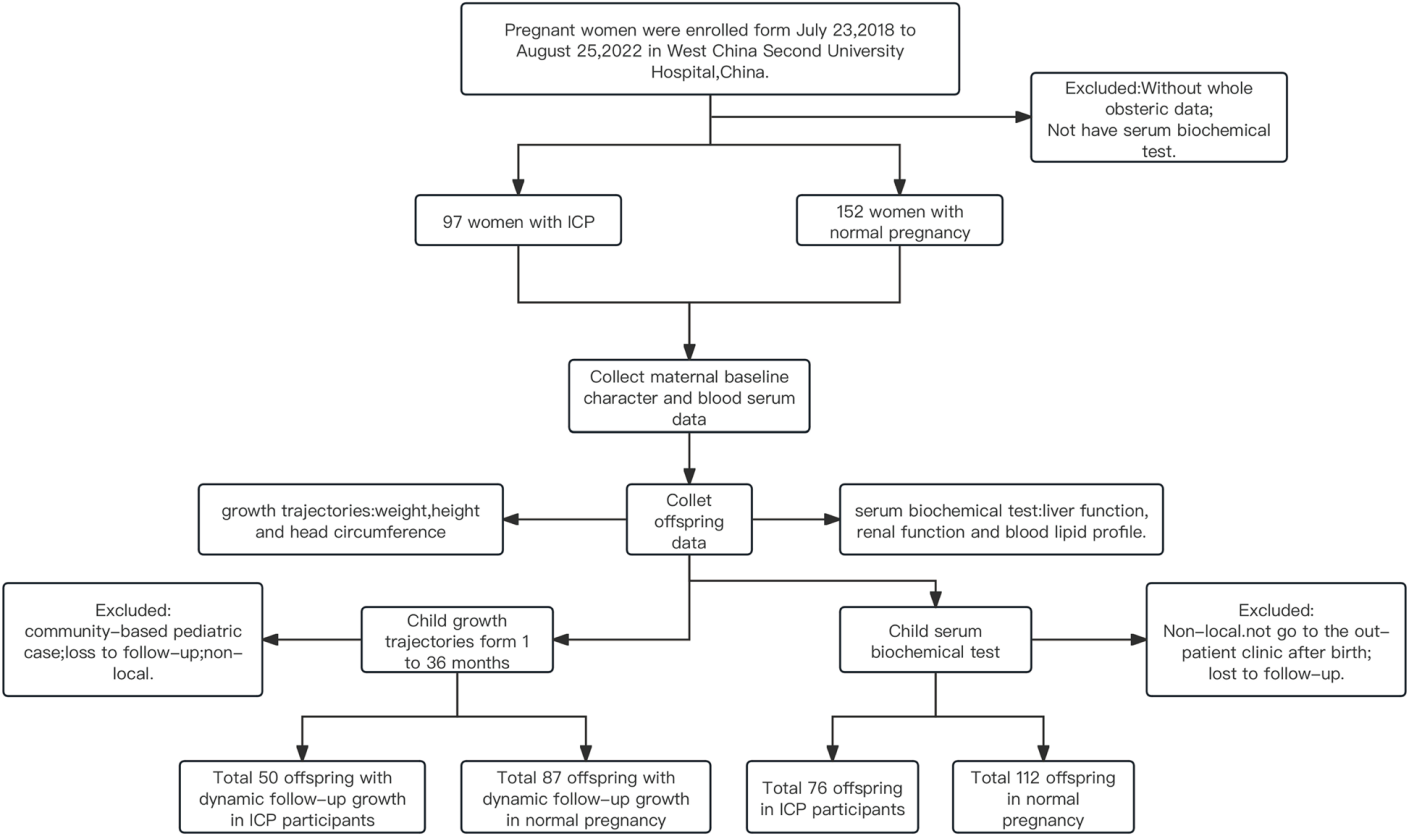
Selection of women and offspring for assessing the impacts of ICP on metabolism and child growth

This nested case‒control cohort study was conducted at West China Second University Hospital from July 23, 2018, to August 25, 2021. A total of 249 pregnant women who had received regular and meticulous prenatal care were enrolled in the study; 97 of these women were diagnosed with ICP (72 singleton and 25 twin pregnancies), and 152 were considered normal controls without any complicated pregnancy (Fig. 1). All of the participants provided written informed consent, and the study was approved by the Research Ethics Committee of the West China Second University Hospital of Sichuan University.

The inclusion criteria for the participants with ICP were a) pregnant women who experienced pruritus without rash and who had fasting TBA levels ≥ 10 µmol/L; b) patients whose gestational age was determined based on the first day of their last menstrual period and/or first trimester ultrasonographic measurements; and c) patients whose complete obstetric and serum data were available.

The exclusion criteria for ICP were HELLP (hemolysis, elevated liver enzymes, and low platelets) syndrome; acute fatty liver during pregnancy; primary biliary cirrhosis; viral hepatitis; and any ultrasound abnormalities.

Non-ICP comparators were healthy pregnancies obtained from the hospital’s birth records between 2018 and 2022. The inclusion criteria for an uncomplicated pregnancy were as follows: a) no history of liver disease; b) complete serologic data throughout the entire pregnancy; and c) prenatal care and delivery at our study hospital.

### Maternal serum data collection

All participants underwent serological testing during the entire pregnancy. Peripheral venous blood was drawn into vacutainer tubes, whereas serum samples for biochemical assays were collected in standard gel separator tubes. Total bile acid (TBA), alanine aminotransferase (ALT), aspartate aminotransferase (AST), γ-glutamyl transferase (GGT), total bilirubin (TBIL), direct bilirubin (DBIL), indirect bilirubin (IBIL), albumin (ALB), albumin/globulin (A/G), alkaline phosphatase (ALP), γ-glutamyl transferase (γ-GT), lactate dehydrogenase (LDH), total protein (TP) and prealbumin (PA) levels were measured using standard laboratory methods. Renal function was assessed based on the levels of uric acid (UA), creatinine (Cr), cystatin C (CYSC), and urea (UN). Lipid profiles were evaluated by measuring total cholesterol (TC), triglyceride (TG), low-density lipoprotein cholesterol (LDL-C), high-density lipoprotein cholesterol (HDL-C), apolipoprotein A1 (APOA1) and apolipoprotein B (APOB), and homocysteine (HCY) levels.

### Child data collection

Our study initially included a total of 122 newborns in the ICP group, including 25 sets of twins, and 152 newborns in the control group. Given the widespread availability of pediatric preventive care in Chinese communities and considering the challenges of follow-up in community settings and with nonlocal women, we ultimately included anthropometric measurements (height, weight, and head circumference) of 50 offspring from ICP participants and 87 offspring from normal pregnancies from our health care clinic's systematic child health care program (Fig. 1). Follow-up for these children began one month postbirth and extended to the longest follow-up date of January 17, 2024. Each child had at least one set of pediatric health records documented during this period.

Weight, length and head circumference were assessed by trained and standardized interviewers. In China, routine pediatric health care typically continues until the age of six, with our longitudinal follow-up data extending up to 36 months. To better evaluate the growth and development of the children, the weight-for-age z score (WAZ), length-for-age z score (LAZ), BMI-for-age z score (BAZ), weight-for-length z score (WLZ) and head-circumference-for-age z score (HCZ) were computed utilizing the reference standards set forth by the World Health Organization (WHO) in 2006 for further analysis, enabling health care professionals to evaluate a child's growth in relation to a globally recognized benchmark [22, 23]. Infants with values outside of the 2006 World Health Organization growth reference standards for WAZ (− 6 to + 5 SD), LAZ (− 6 to + 6 SD), WLZ (− 5 to + 5 SD), BAZ (-5 TO + 5 SD) and HCZ (-5 to + 5 SD) were considered invalid and excluded from analysis.

The serological data for the children were obtained from the hospital's laboratory information system following a process similar to that used for the mothers. In total, 76 offspring of ICP patients and 112 offspring of non-ICP women had at least one serological data point (liver function, renal function and blood lipid profile).

### Monitoring pregnancy outcomes and child growth

Adverse neonatal outcomes included neonatal pneumonia, hypoproteinemia, neonatal hypoglycemia, neonatal anemia, pathological jaundice of the newborn (PJON), neonatal apnea, meconium staining of amniotic fluid (MSAF), neonatal respiratory distress syndrome (NRDS), small for gestational age (SGA) and neonatal asphyxia.

Preterm birth was defined as a liveborn infant with a gestational age < 37 completed weeks. Small for gestational age (SGA) was defined as a birthweight < 10th percentile. PJON refers to a condition characterized by elevated levels of bilirubin in a neonate's blood, manifesting within the first 24 h postbirth. NRDS is characterized by progressive respiratory difficulty, cyanosis, expiratory groaning, inspiratory retraction signs, and respiratory failure manifesting shortly after birth.

Infants born to mothers with known outcomes, baseline characteristics and at least one type of pediatric data were included in the anthropometry analyses. Children were defined as having abnormal development if they had at least one instance of LAZ, WAZ, BAZ, WLZ or HCZ score below -2 [24] or if they were diagnosed with global developmental delay language in outpatient medical records. Underweight was defined as a WAZ < -2, and stunting was defined as a LAZ < -2 according to the 2006 WHO growth standards [22, 24].

### Statistical methods

Data were verified, filtered, and subsequently analyzed using R version 4.3.1. For analyzing categorical variables, chi-square tests were employed. Continuous variables that were normally distributed were described using the mean and standard deviation (mean [SD]) and were analyzed with independent sample t tests. Conversely, variables exhibiting skewed distributions were described using medians and interquartile ranges (medians [IQRs]) and analyzed using the Mann‒Whitney U test. Growth indices such as LAZ, WAZ, WLZ, BAZ, and HCZ were calculated based on the 2006 WHO growth reference values using the ‘anthro’ package in R, and stratified analysis was conducted based on month. Correlation analyses were performed using the ‘state’ package to examine pairwise associations among various variables of interest. This approach, utilizing pairwise complete observations, ensures that each correlation coefficient is based on the maximum available data for each variable pair. Spearman correlation analysis was employed to identify relationships between the maximum levels of maternal LDH and TBA and indicators of abnormal child development, including LAZ and WAZ. We employed univariate logistic regression to determine the odds ratio (OR) for the likelihood of abnormal development in offspring and examined the association between elevated levels of TBA and LDH in the two groups. Significance was ascribed to two-tailed p values less than 0.05.

## Results

### Clinical details of the participants

The characteristics of the women enrolled in the study are detailed in Table 1. No significant difference was observed in maternal age at delivery or pregestational BMI between the groups. Approximately 8.3% of women in the ICP group reported a history of ICP, whereas none of the women in the non-ICP comparison group reported a history of ICP. The participants with ICP exhibited a greater incidence of preterm birth (62% vs. 85%, *p* < 0.05) and cesarean Sect. (81.4% vs. 30.9%, *p* < 0.05). Significant differences were also noted in birth weight, birth height, and placental weight between offspring of mothers with ICP and offspring of healthy mothers, consistent with our previous study in a mouse model of ICP induced by 17α-ethynyl estradiol [25].

**Table 1:** Baseline characteristics of the included women

**The baseline characteristics of mothers**			
***Between ICP and Normal Pregnancy***			
	ICP *N *= 97	Normal *N *= 152	*P*-Value^*1*^
**Maternal Age At Delivery, year, Median (IQR)**	31(5)	30(6)	
**Inch, N (%)**	49(50.5)	0(0.0)	
**Preterm birth, N(%)**	60(62)	129(85)	<0.001
**Gestational weeks, Median (IQR)**	37(3)	39(3)	<0.001
**Height, cm, Median (IQR)**	160(6)	159(6)	
**Gravidity, Mean (SD)**	2.3(1.35)	1.6(0.87)	<0.05
**Parity, Median (IQR)**	1.3(0.51)	1.1(0.34)	
**CS**	79(81.4)	47(30.9)	
**VD**	18(18.6)	105(69.1)	
**Singleton N(%)**	72(74.2)	152(100)	
**Twin N(%)**	25(25.8)	0(0)	
**Pregestational BMI, kg/m2,(IQR)**	20.7(3.37)	20.1(3.70)	
**Weight Gain, kg, Median(IQR)**	11.0(5)	13.5(5)	<0.05

*Abbreviation*: CS cesarean, VD vaginal delivery

^1^*P*-Values are displayed only when statistical significance is achieved

There were 122 live births recorded in the ICP group and 152 live births in the control (Con) group. Of these, 68 neonates born to mothers diagnosed with ICP were admitted to the neonatal care unit (NCU) in contrast to 82 offspring from pregnancies without complications (*P* = 0.862). A greater proportion of newborns from mothers with ICP were admitted to the neonatal intensive care unit (NICU) (19.6% vs. 3.3%, *P* < 0.001). The pregnancy outcomes for newborns of mothers with ICP and those of mothers with normal pregnancies are detailed in Additional file 1: Supplementary Table 1. Compared with newborns from normal pregnancies, newborns from mothers with ICP exhibited increased rates of MASF, neonatal pneumonia, hypoproteinemia, hypoglycemia, neonatal anemia, PJON, NRDS, neonatal apnea, neonatal respiratory failure, SGA, and asphyxia. However, only hypoglycemia reached statistical significance (*P* = 0.029).

### Comparison of maternal liver function, renal function and blood lipid profiles between the ICP and Con groups

We collected serum biomarkers, including liver function, renal function and blood lipids, throughout pregnancy and recorded the maximum values of these serum biomarkers for further study. The results are shown in Table 2.

**Table 2:** Comparison of liver function, renal function and blood lipid profiles between mothers with ICP and normal controls

The baseline characteristics of maternal serum marker			
***Between ICP and Normal Pregnancy***			
**Description**	ICP *N* = 97	Normal *N* = 152	*P*-Value^*1*^
**Liver Function**			
TBA, Median(IQR)	20.1(18.7)	2.5(2.2)	< 0.001*
ALT, Median(IQR)	178.0(223.5)	26.0(16.3)	< 0.001*
AST, Median(IQR)	103.0(146.0)	26.0(10.3)	< 0.001*
TB, Mean(SD)	16.1(8.9)	11.6(3.6)	< 0.001*
IBIL, Mean(SD)	10.9(4.7)	8.85(2.9)	< 0.001*
DBIL, Mean(SD)	4.9(4.50)	3.1(1.2)	< 0.001*
TP, Median(IQR)	72.9(6.8)	71.85(5.6)	0.095
ALB, Mean(SD)	45.2(3.7)	44.5(3.5)	0.121
A/G, Median(IQR)	1.7(0.3)	1.7(0.2)	0.141
ALP, Median(IQR)	233.0(108.5)	160.5(82.3)	< 0.001*
γ-GT, Median(IQR)	53.0(62.5)	19(12.3)	< 0.001*
LDH, Median(IQR)	247.5(123.0)	181.0(35.5)	< 0.001*
PA, Median(IQR)	257.5(55.3)	246.5(48.5)	0.039*
**Renal Function**			
Cr, Median(IQR)	51.5(14.0)	48.0(9.0)	0.003*
UA, Median(IQR)	395.5(156.5)	320.0(84.5)	< 0.001*
UN, Median(IQR)	4.8(2.2)	3.8(1.2)	< 0.001*
CYSC, Median(IQR)	1.5(0.7)	1.1(0.29)	< 0.001*
**Blood Lipid**			
TC, Median(IQR)	6.2(2.3)	6.3(1.5)	0.425
TG, Median(IQR)	3.8(2.7)	3.3(1.9)	0.016*
HDL-C, Median(IQR)	1.8(0.7)	2.0(0.6)	< 0.001*
LDL-C, Median(IQR)	4.3(1.9)	3.3(1.3)	< 0.001*
APOA1, Median(IQR)	1.8(0.5)	2.1(0.4)	< 0.001*
APOB, Median(IQR)	1.4(0.5)	1.1(0.3)	< 0.001*
HCY, Median(IQR)	7.3(2.2)	6.5(2.3)	< 0.001*

Mentioned: All Serum Markers are Represented by the Highest Values Measured During Pregnancy

*Abbreviation*: *TBA* total bile acid, *ALT* alanine aminotransferase, *AST* aspartate aminotransferase, *GGT* γ-glutamyl transferase, *TBIL* total bilirubin, *DBIL* direct bilirubin, *IBIL* indirect bilirubin, *TP* total protein, *ALB* albumin, *A/G* albumin/globulin, *ALP* alkaline phosphatase, *γ-GT* γ-Glutamyl Transferase, *LDH* lactate dehydrogenase, *PA* prealbumin, *UA* uric acid, *Cr* creatinine, *CYSC* cystatin C, *UN* urea, *TC* total cholesterol, *TG* triglycerides, *LDL* low-density lipoprotein cholesterol C, *HDL-C* high-density lipoprotein cholesterol C, *APOA1* apolipoproteins A1, *APOB* apolipoproteins B, *HCY* homocysteine

^1^ The '*' symbol denotes *P* values < 0.05 and are statistically significant

In patients with ICP, we observed a marked increase in the TBA level (20.1 vs. 2.5) and significant differences in the ALT and AST levels, both of which are indicators of hepatic injury. The maternal concentrations of TB, IBIL, and DBIL were significantly greater in the group of pregnant women with ICP than in those with normal pregnancies. Although TP and ALB were greater in the ICP group, the differences did not reach statistical significance. ALP, γ-GT, LDH, and PA levels were also significantly elevated in the ICP groups.

It is widely known that the ICP is associated with impaired liver function, but research on the detection of renal function is rare. Renal function, which is less frequently associated with ICP in research, revealed significant differences in Cr, UA, UN, and CYSC levels (*P* < 0.05) in our study (Table 2).

Furthermore, our analysis indicates a potential link between ICP and maternal dyslipidemia, with ICP patients showing significantly higher TG and altered HDL-C and LDL-C levels, consistent with our previous findings [21]. TC levels were not significantly different between the two groups (median 6.2 vs. 6.3). APOB (median 1.4 vs. 1.1) and HCY (median 7.3 vs. 6.5) concentrations were significantly increased in the ICP group, whereas APOA1 (median 1.8 vs. 2.1) levels were significantly reduced (Table 2).

### Comparison of offspring liver function, renal function and blood lipid profiles between the ICP and Con groups

Our findings (Table 3) indicate a significant increase in ALT concentrations in offspring from the ICP group (median 21 vs. 16, *P* < 0.05), with no notable differences in ALT, TB, IBIL, TP, ALB, A/G, LDH, γ-GT, or PA levels. Despite maternal renal impairment, biomarkers of renal function in offspring serum showed no differences between the groups. Lipid profile analysis revealed higher median HDL-C (1.05 vs. 0.90, *P* < 0.05), LDL-C (2.0 vs. 1.3, *P* < 0.05), and APOB (0.59 vs. 0.38, *P* < 0.05) levels in offspring from ICP-affected pregnancies (Table 3), suggesting potential long-term metabolic implications.

**Table 3:** Comparison of liver function, renal function and blood lipid profiles between the offspring of the two groups

The baseline characteristics of offspring serum marker			
***Between ICP and Normal Pregnancy***			
**Description**	ICP	Normal	*P*-Value^*1*^
**Liver Function**	*N* = 76	*N* = 112	
TBA, Median(IQR)	10.4(14.7)	8.2(4.5)	0.12
ALT, Median(IQR)	21(18.0)	16(15.0)	0.02*
AST, Median(IQR)	53(29.0)	52(32.5)	0.56
TB, Median(IQR)	209.4(227.9)	214.9(188.5)	0.48
IBIL, Median(IQR)	194.8(192.4)	199.1(213.1)	0.05
DBIL, Median(IQR)	14.7(14.5)	19.4(14.3)	0.04*
TP, Median(IQR)	53.3(9.5)	52.8(7.9)	0.99
ALB, Median(IQR)	36(6.7)	35.1(5.4)	0.44
A/G, Median(IQR)	2(0.4)	2(0.3)	0.47
LDH, Median(IQR)	440.5(296.5)	439.0(233.0)	0.77
γ-GT, Median(IQR)	118(115.5)	115(96.5)	0.78
PA, Median(IQR)	119.5(100.8)	92.0(64.5)	0.11
**Renal Function**	*N* = 66	*N* = 68	
Cr, Median(IQR)	40(36.5)	42.5(25.5)	0.64
UA, Median(IQR)	282(188.0)	261(132.2)	0.12
UN, Median(IQR)	3.80(2.1)	3.52(2.2)	0.31
CYSC, Median(IQR)	1.53(0.6)	1.59(0.3)	0.48
**Blood Lipid**	*N* = 47	*N* = 37	
TC, Median(IQR)	3.54(1.1)	3.07(1.6)	0.79
TG, Median(IQR)	1.20(0.7)	1.18(0.9)	0.67
HDL-C, Median(IQR)	1.05(0.5)	0.90(0.4)	0.03*
LDL-C, Median(IQR)	2.0(1.3)	1.3(1.3)	0.01*
APOA1, Median(IQR)	1.09(0.4)	0.95(0.4)	0.14
APOB, Median(IQR)	0.59(0.3)	0.38(0.3)	0.04*
HCY, Median(IQR)	6.10(1.6)	6.28(2.6)	0.84

Mentioned: All Serum Markers are Represented by the Highest Values

*Abbreviation*: *TBA* total bile acid, *ALT* alanine aminotransferase, *AST* aspartate aminotransferase, *GGT* γ-glutamyl transferase, *TBIL* total bilirubin, *DBIL* direct bilirubin, *IBIL* indirect bilirubin, *TP* total protein, *ALB* albumin, *A/G* albumin/globulin, *ALP* alkaline phosphatase, *γ-GT* γ-glutamyl transferase, *LDH* lactate dehydrogenase, *PA* prealbumin, *UA* uric acid, *Cr* creatinine, *CYSC* cystatin C, *UN* urea, *TC* total cholesterol, *TG* triglycerides, *LDL* low-density lipoprotein cholesterol C, *HDL-C* high-density lipoprotein cholesterol C, *APOA1* apolipoproteins A1, *APOB* apolipoproteins B, *HCY* homocysteine

^1^The '*' symbol denotes *P* values < 0.05 and are statistically significant

### Stratified analysis of offspring LAZ, WAZ, WLZ, BAZ and HCZ by month

To further explore the long-term effects of ICP on offspring growth development compared with those of normal pregnancies, we compared LAZ, WAZ, WLZ, BAZ, and HCZ based on the timing of pediatric health care visits (Table 4). In the 1- to 3-month age range, median BAZ values were similar between the groups. However, LAZ, WAZ, WLZ and HCZ values were significantly different between the ICP and Con groups (*P* < 0.01). In the following three-month period, significant differences were observed in LAZ (median -0.42 vs. 0.12), WAZ (median -0.12 vs. 0.49), WLZ (median 0.31 vs. 0.61), BAZ (median 0.16 vs. 0.57), and HCZ (median -0.21 vs. 0.32) values between the offspring of ICP-affected pregnancies and the normal control group. In the 7- to 9-month age range, evidence of differences in LAZ (median -0.19 vs. 0.28), WAZ (median 0.12 vs 0.47), and HCZ (median 0.04 vs. 0.5) values were found between the two groups, whereas BAZ (median 0.31 vs. 0.42) and WLZ (0.39 vs. 0.52) values were similar. At 10–12 months, there was little evidence of a difference in the BAZ (median 0.26 vs. 0.57), while statistically significant differences were observed in the LAZ, WAZ, WLZ, and HCZ (*P* < 0.05). Between 1 and 2 years, LAZ, WAZ, WLZ, BAZ, and HCZ values showed minimal differences. Similarly, in the 2- to 3-year age range LAZ, WLZ, BAZ, and HCZ values were comparable between the groups. However, WAZ levels displayed evidence of difference, with ICP participants showing a median of -0.46 compared with 0.22 in the normal control group. Intriguingly, after the exclusion of a child with global developmental delay from the ICP group, WAZ levels were found to be similar between the two groups.

**Table 4:** Comparison of growth velocity between children in the ICP and normal groups based on month

The comparison of offspring growth velocity					
***Between ICP and Normal Pregnancy***					
**Month**		ICP	Normal	P-Value^*1*^	
**1–3**	LAZ, Mean(SD)	-0.92(1.29)	-0.28(0.98)	0.002*	
	WAZ, Mean(SD)	-0.73(1.28)	-0.14(0.96)	0.006*	
	WLZ, Mean(SD)	0.12(0.95)	0.21(0.94)	0.686	
	BAZ, Mean(SD)	-0.32(1.08)	0.00(0.95)	0.072	
	HCZ, Mean(SD)	-0.52(1.07)	0.17(1.55)	0.001*	
**4–6**	LAZ, Mean(SD)	-0.42(1.14)	0.12(1.04)	< 0.001*	
	WAZ, Mean(SD)	-0.12(1.10)	0.49(1.06)	< 0.001*	
	WLZ, Mean(SD)	0.31(0.93)	0.61(0.98)	< 0.024*	
	BAZ, Mean(SD)	0.16(0.96)	0.57(0.99)	0.002*	
	HCZ, Mean(SD)	-0.21(1.05)	0.32(1.02)	< 0.001*	
**7–9**	LAZ, Mean(SD)	-0.19(1.03)	0.28(0.97)	0.004*	
	WAZ, Mean(SD)	0.12(1.09)	0.47(0.99)	0.042*	
	WLZ, Mean(SD)	0.39(0.93)	0.52(0.85)	0.377	
	BAZ, Mean(SD)	0.31(0.93)	0.42(0.84)	0.439	
	HCZ, Mean(SD)	0.04(1.01)	0.50(0.91)	0.005*	
**10–12**	LAZ, Mean(SD)	-0.37(1.60)	0.16(0.90)	0.011*	
	WAZ, Mean(SD)	-0.02(1.01)	0.51(0.85)	0.007*	
	WLZ, Mean(SD)	0.24(0.88)	0.61(0.81)	0.042*	
	BAZ, Mean(SD)	0.26(0.86)	0.57(0.81)	0.084	
	HCZ, Mean(SD)	0.00(0.92)	0.37(0.77)	0.036*	
**13–18**	LAZ, Mean(SD)	-0.31(0.99)	-0.01(0.97)	0.110	
	WAZ, Mean(SD)	-0.10(0.94)	0.03(0.93)	0.375	
	WLZ, Mean(SD)	0.05(0.85)	0.07(0.82)	0.869	
	BAZ, Mean(SD)	0.10(0.82)	0.07(0.76)	0.812	
	HCZ, Mean(SD)	0.11(0.84)	0.30(0.89)	0.222	
**18–24**	LAZ, Mean(SD)	-0.11(0.94)	0.15(0.91)	0.208	
	WAZ, Mean(SD)	-0.10(0.89)	0.21(0.84)	0.120	
	WLZ, Mean(SD)	-0.06(0.86)	0.18(0.81)	0.218	
	BAZ, Mean(SD)	-0.03(0.86)	0.18(0.82)	0.261	
	HCZ, Mean(SD)	0.35(0.77)	0.36(0.92)	0.964	
**24–36**	LAZ, Mean(SD)	-0.02(0.89)	0.45(0.92)	0.151	
	WAZ, Mean(SD)	-0.46(0.79)	0.22(1.02)	0.049*	
	WLZ, Mean(SD)	-0.68(0.91)	-0.06(1.03)	0.085	
	BAZ, Mean(SD)	-0.70(0.94)	-0.10(1.03)	0.098	
	HCZ, Mean(SD)	0.35(0.79)	0.42(1.11)	0.853	

*Abbreviation*: *LAZ* length-for-age z score, *WAZ* weight-for-age z score, *WLZ* weight-for-length z score, *BAZ* BMI-for-age z score, *HCZ* head-circumference-for-age z score

^1^The '*' symbol denotes *P* values < 0.05 and are statistically significant

## The diagnosis of abnormal development

In the ICP group, one child was diagnosed with global developmental delay, and two children exhibited language delays. In the control group, one child experienced language delay (*P* = 0.554). A significant difference in the incidence of stunting was noted between the two groups (12 vs. 6, *P* = 0.004). Conversely, the difference in the underweight rate between the two groups was not statistically significant (8 vs. 7, *P* = 0.166). Furthermore, the offspring in the ICP group demonstrated a greater rate of abnormality in HCZ (P = 0.034) (Additional file 1: Supplementary Table 2).

## Correlation analysis between maternal serum biomarkers and offspring serum biomarkers

**Fig. 2:**
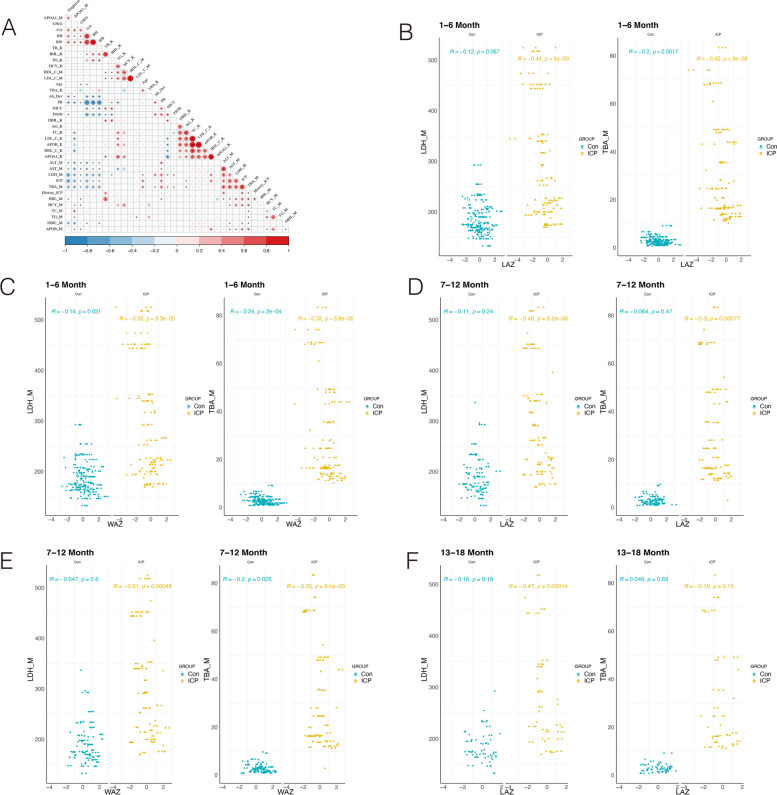
Correlation analysis of baseline characteristics, serological test results and offspring growth indicators between pregnant women with ICP and pregnant healthy controls. **A** Correlations among baseline characteristics, serological tests and abnormal offspring development. “*” represents *P* < 0.05. Red indicates a positive correlation, and blue signifies a negative correlation. The intensity of the color directly correlates with the strength of the relationship. Scatter plots depicting the correlation between maximum maternal LDH levels (left) and maximum maternal TBA levels (right) and offspring LAZ or WAZ at different time intervals. LAZ (**B**) and WAZ (**C**) values in offspring during the 1- to 6-month age range, LAZ (**D**) and WAZ (**E**) values in offspring during the 7- to 12-month age range, and LAZ (**F**) values in offspring during the 13- to 18-month age range. WAZ values in the 7- to 12-month age range, LAZ values in the 13- to 36-month age range, and WAZ values in the 13- to 36-month age range can be found in Additional file 2: Figure S1. Green represents the control group, and yellow represents the ICP group. Correlation coefficients (R) and *p* values are provided for each group to indicate the strength and significance of the correlations. Abbreviations: GWG, gestational weight gain; BH, birth height; BW, birth weight; K, child; M, mother; Ab_Dev, abnormal development (LAZ, WAZ, WLZ, BAZ or HCZ < -2); PB, preterm birth; NICU, neonatal intensive care unit; PJON, pathological jaundice of newborn. LAZ, length-for-age score. WAZ, weight-for-age z score. All serum markers are represented by the highest values

To investigate the associations between abnormal offspring development and serum biomarkers, we conducted a correlation analysis. The results (Fig. 2A) indicate that abnormal development in offspring is negatively associated with singleton birth, maternal APO-A1 levels, gestational week, birth weight, and birth height (*P* < 0.05). Conversely, factors such as preterm birth, PJON, elevated maternal TBA levels, and maternal LDH levels are associated with an increased incidence of abnormal offspring development (*P* < 0.05).

Furthermore, our findings revealed that maternal maximum TBA levels were positively correlated with markers of liver function, including ALT, AST, DBIL, IBIL, and GGT (*P* < 0.05). TBA concentrations were also correlated with maternal blood lipid indicators such as TC, TG, and APOB (*P* < 0.05). However, maternal APOA1 levels were negatively correlated with TBA levels (*P* < 0.05) (Fig. 2A).

## The relationship between maternal TBA levels and offspring development

To further elucidate the relationship between serum markers and the abnormal growth development of offspring, we analyzed data concerning offspring health care alongside maternal TBA and LDH levels.

During the first 12 months, positive correlations were observed between the maximum maternal TBA and LDH levels and decreased LAZ and WAZ in both the ICP and normal groups. In the analysis of anthropometric data from 1 to 6 months, a statistically significant negative correlation was noted between LAZ scores and maternal TBA levels in both the ICP and normal groups (Fig. 2B, C). Specifically, maternal TBA levels were moderately negatively correlated with LAZ values, with a correlation coefficient of -0.42 in the ICP group (Fig. 2B). In the control group, the correlation coefficient was -0.2. WAZ scores also demonstrated a negative correlation with TBA concentration in both groups (Fig. 2C).

From 7 to 12 months, both offspring LAZ (R = -0.35) and WAZ (R = -0.3) scores were negatively correlated with maternal TBA levels in the ICP group, whereas only WAZ scores showed a statistical difference in the normal group (Fig. 2D, E). However, at 13 to 24 months and 25 to 36 months, no significant differences were found between the maximum maternal TBA levels and LAZ or WAZ scores in either group (Fig. 2E, Additional file 2: Supplementary Fig. 1).

Moreover, univariate logistic regression analysis identified TBA levels as an independent risk factor for the abnormal development of offspring (Additional file 1: Supplementary Table 3). TBA levels greater than 10 μmol/L and lesser than 40 μmol/L were associated with a 2.05-fold increase in the odds of abnormal development in offspring; however, this association did not reach statistical significance (OR = 2.05, 95% CI = 0.71 to 5.88, *P* value = 0.183). A more pronounced effect was observed for TBA levels exceeding 40 μmol/L, which were associated with a 5.85-fold increase in the odds of abnormal development (OR = 5.85, 95% CI = 1.55 to 22.04, *P* value = 0.009).

## The relationship between maternal LDH levels and offspring development

Building upon previous analysis, we also detected relationships between maternal LDH levels and LAZ and WAZ values. From 1 to 6 months and 7 to 12 months (Fig. 2B-E), maternal LDH levels were significantly negatively correlated with LAZ and WAZ values in the ICP group (*P* < 0.05). However, in the Con group, minimal differences were observed. From 13 to 18 months and 19 to 24 months (Fig. 2E, Additional file 2: Supplementary Fig. 1), a significant difference was observed in the relationship between maternal LDH concentration and LAZ and WAZ scores within the ICP group. However, in normal pregnancies, this relationship did not reach statistical significance.

From 25 to 36 months, no evidence was found to suggest a relationship between maternal LDH and LAZ concentrations in either group (Additional file 2: Supplementary Fig. 1). Additionally, in both the normal and the ICP groups, WAZ was not associated with the highest maternal LDH levels.

On the other hand, univariate logistic regression analysis demonstrated a statistically significant association between LDH levels and the risk of abnormal offspring development in the ICP group (OR = 1.004, 95% CI = 1.00 to 1.01; *P* = 0.012) (Additional file 1: Supplementary Table 3). This indicates a modest but significant increase in risk with increasing LDH levels. However, LDH levels were not significantly associated with the risk of abnormal development in offspring from the normal group (OR = 1.003, 95% CI = 0.98 to 1.01, *P* = 0.667) (Additional file 1: Supplementary Table 4).

## Discussion

In our nested case‒control cohort study, we found that offspring of ICP-affected pregnancies show impaired growth and development. However, after 12 months, no differences in LAZ, WLZ, BAZ or HCZ values were observed. Moreover, ICP mothers were more likely to have renal function and blood lipid abnormalities in addition to liver function abnormalities; however, only minimal differences in offspring metabolism were observed.

Elevated bile acids due to liver dysfunction can contribute to renal impairment by inducing vasoconstriction, oxidative stress and inflammation in renal tissues [26, 27]. Elevated bile acids can impair kidney function in patients with chronic renal failure [26]. The relationship between bile acids and lipid metabolism is complex and plays a crucial role in the regulation of lipid homeostasis and cardiovascular health. Bile acids not only are essential for the digestion and absorption of lipids but also regulate lipid levels through signaling pathways such as FXR (Farnesoid X Receptor) and TGR5 [28], which influence various aspects of lipid metabolism, including cholesterol homeostasis, triglyceride levels, and lipoprotein synthesis, contributing to the maintenance of overall metabolic health [18, 29]. In our study, offspring LDL-C, HDL-C and APOB values were significantly increased in the ICP group.

Specifically, differences in liver function and blood lipid levels were noted between children born to mothers diagnosed with ICP and those born to mothers with normal pregnancies. The resolution of bile acid metabolism and dyslipidemia in newborns of mothers with ICP after birth could be attributed to their own metabolic adjustments. Research highlights significant changes in bile acid metabolism during pregnancy progression in ICP patients compared to normal pregnant women, indicating that altered BA metabolism in ICP patients could impact fetal development [30]. However, after birth, the neonatal liver and gastrointestinal system begin functioning independently, potentially normalizing these metabolic pathways​. Additionally, the gut microbiota plays a crucial role in BA metabolism [31] and is significantly altered postnatally as newborns are exposed to the external environment and begin feeding. Hence, abnormal maternal metabolism may affect fetal outcomes. Abnormal maternal lipid levels are associated with the risk of small for gestational age (SGA) infants [32]. These changes can significantly impact fetal development and lead to complications such as fetal growth restriction and preterm birth [33]. However, research related to the abnormal growth and development of ICP-affected offspring has not been reported.

To the best of our knowledge, we are the first to trace the development trajectory of children born to mothers with ICP versus those with normal pregnancies from birth to a maximum of 36 months. From 1 to 12 months, LAZ, WAZ and HCZ values of the offspring in the ICP group significantly decreased, indicating relatively poor development. Low birth weight (LBW) was used to identify infants born with insufficient growth because this metric exclusively relies on weight at birth [34]. Our study revealed that neonatal birth weight and birth height were lower in the ICP group than in the normal pregnancy group, which may partly explain the difference in birth age. However, between 12 and 36 months, similar values were observed between the two groups, suggesting that the impact of maternal TBA levels on offspring anthropometric measures may diminish as the child ages beyond the first year of life. WAZ values were slightly different between the ICP group and the normal group at 25 to 36 months, and the P value obtained was potentially influenced by the diagnosis of global developmental delay in one child (Supplementary Fig. 1). Global developmental delay (GDD) is defined as a significant delay in two or more developmental domains, including gross and fine motor skills, speech and language, cognition, social and personal skills, and activities of daily living, in children under the age of 5 years. After removing the children of mothers with ICP who were diagnosed with GDD, WAZ reanalysis results showed no significant difference.

Catch-up growth (CUG) is commonly defined as a height velocity that is more rapid than average for individuals of a comparable age or maturity, allowing them to 'catch up' to the growth trajectory of their full-term peers [34]. The difference in growth velocity suggested the possibility of catch-up growth in the offspring of mothers with ICP. A potential mediator linking ICP to offspring growth and development is preterm birth. A study revealed that over 80% of late preterm and small for gestational age (SGA) infants exhibited catch-up growth in weight and length at 3 and 6 months of corrected age. However, this growth trajectory seemed to slow after the first 6 months, particularly when solid foods were introduced, which could cause feeding challenges and potentially affect growth [35].

Adequate and appropriate nutritional support is crucial for catch-up growth [36]. Advocating for exclusive breastfeeding and implementing strategies for the prevention and prompt management of pneumonia are also critical interventions that may contribute to optimizing child growth outcomes [37]. Preterm infants undergo various metabolic adaptations after birth to support catch-up growth. For neonates born to mothers with ICP, the reasons for catch-up growth may be similar. After birth, the resolution of exposure to elevated maternal bile acids, along with appropriate nutritional and medical support, enable these infants to undergo catch-up growth. Environmental factors may also influence catch-up growth [32].

Interestingly, initial rapid catch-up growth leads to morphological abnormalities in pancreatic islets and fibrosis, which are linked to alterations in the expression of cell adhesion-related proteins. This process subsequently results in glucose intolerance and dyslipidemia in male rats [38]. Although the offspring of individuals with ICP may achieve relatively normal growth trajectories, the long-term impacts of ICP on these offspring necessitate ongoing surveillance. Although catch-up growth is possible and does occur in infants with ICP, it is variable and may be influenced by a range of factors, including nutrition, socioeconomic status, gestational age at birth, and health care practices. Monitoring the growth of ICP infants closely and providing guidance on nutrition and care to support optimal growth outcomes are needed.

Growth stunting is associated with concurrent and longer-term deficits in cognition, behavior, motor skills, and school performance [39]. The HCZ is generally regarded as an important indicator of neurodevelopmental progress in infants and young children. A smaller than average HCZ potentially indicates issues with brain development, potentially leading to cognitive, motor, and language development delays [40, 41]. In our study, the incidence of stunting (LAZ < -2) and abnormal HCZ (HCZ < -2) significantly increased in the ICP group. Interestingly, a cohort study revealed that offspring of an ICP-affected pregnancy are more likely to be diagnosed with neurodevelopmental conditions [14]. Although catch-up growth was noted after the age of one and offspring from the ICP cohort displayed no significant developmental disparities compared to those from the control group, the ICP group had relatively lower HAZ, WAZ, WLZ, BAZ and HCZ values and a significantly greater incidence of stunting and abnormal HCZ. These findings indicate an elevated risk of developmental anomalies despite catch-up growth and the need for dynamic monitoring of the offspring of ICP participants.

In liver diseases, particularly cholestasis, LDH and TBA serve as important biomarkers. LDH levels indicate liver cell damage because LDH is released into the bloodstream during cell injury. ICP patients exhibit elevated LDH levels in serum liver function tests [42]. Elevated TBA levels are more specific to cholestasis, reflecting impaired bile flow and accumulation of bile acids in the liver and blood. In our study, we found that maximum maternal LDH and TBA levels were positively related to lower LAZ and WAZ values from 1 to 12 months. Total bile acid (TBA) levels greater than 40 µmol/L are associated with severe intrahepatic cholestasis of pregnancy (ICP). This condition not only poses significant maternal risks but also increases the likelihood of developmental abnormalities in offspring. Close monitoring of these biomarkers in mothers could be crucial, emphasizing the importance of proactive management in cases of liver disease and cholestasis to mitigate adverse outcomes in offspring.

A key innovation from this nested case‒control cohort study is that we dynamically tracked the pediatric health care data in offspring from 1 month to a maximum of 36 months. In addition, we performed serological tests in the ICP groups and analyzed the relationship between offspring growth and maternal serum biochemistry markers.

A significant limitation of our study is the incomplete collection of pediatric health data due to the difficulty in gathering community health records and the nonlocal status of some women, resulting in a relatively small sample size of offspring. A larger, multicenter population-based cohort is needed to track the development of offspring in ICP groups over a more extended period of time and to explore the potential underlying mechanisms of this relationship.

## Conclusion

The maximum values of maternal liver function, renal function, and blood lipid tests were significantly greater in the ICP groups, with only slight differences detected in the offspring of women with ICP. From 1 to 12 months, offspring from pregnant women with ICP exhibit lower growth velocities. However, after 12 months, these children demonstrate catch-up growth until 3 years of age. Elevated LDH and TBA levels in women with ICP are associated with an increased risk of diminished LAZ and WAZ values during the first 12 months. Additionally, offspring with ICP have a greater incidence of stunting and abnormal HCZ (HCZ < -2).

### Supplementary Information

Additional file 1: Supplementary Table 1. Comparison of the Neonatal Baseline Characteristics between Pregnant Patients with ICP and Patients with Normal Pregnancies. Supplementary Table 2. Diagnosis of abnormal development in offspring between the ICP group and the normal group. Supplementary Table 3. Logistic regression of abnormal development in offspring in the ICP group. Supplementary Table 4. Logistic regression of abnormal offspring development in the normal group.

Additional file 2: Figure 1. Correlation Analysis of Serological Markers in Mothers from the ICP and Normal Groups with Child Development Indicators. Scatter plots depicting the correlation between maximum maternal LDH levels (left) and maximum maternal TBA levels (right) and offspring LAZ or WAZ values at different time intervals. WAZ (A) values in offspring during the 13- to 18-month age range, LAZ (B) and WAZ (C) values in offspring during the 19- to 24-month age range, and LAZ (D) and WAZ (E) values in offspring during the 25- to 36-month age range. Green represents the control group, and yellow represents the ICP group. Correlation coefficients (R) and p values are provided for each group to indicate the strength and significance of the correlations. Abbreviations: GWG, gestational weight gain; BH, birth height; BW, birth weight; K, child; M, mother; Ab Dev, abnormal development (LAZ, WAZ, WLZ, BAZ or HCZ <-2); PB, preterm birth; NICU, neonatal intensive care unit; PJON, pathological jaundice of newborn. LAZ, length-for-age z score. WAZ, weight-for-age z score.

## Data Availability

The datasets used and/or analysed during the current study are available from the corresponding author on reasonable request.

## Abbreviations

NCU: Neonatal care unit
NICU: Neonatal intensive care unit
PJON: Pathological jaundice of the newborn
MSAF: Meconium staining of amniotic fluid
NRDS: Neonatal respiratory distress syndrome
SGA: Small for gestational age
TBA: Total bile acid
ALT: Alanine aminotransferase
AST: Aspartate aminotransferase
GGT: γ-Glutamyl transferase
TBIL: Total bilirubin
DBIL: Direct bilirubin
IBIL: Indirect bilirubin
ALB: Albumin
A/G: Albumin/globulin
ALP: Alkaline phosphatase
γ-GT: γ-Glutamyl transferase
LDH: Lactate dehydrogenase
TP: Total protein
PA: Prealbumin
TBA: Total bile acid
UA: Uric acid
Cr: Creatinine
CYSC: Cystatin C
UN: Urea
TC: Total cholesterol
TG: Triglycerides
LDL: Low-density lipoprotein cholesterol C
HDL-C: High-density lipoprotein cholesterol C
APOA1: Apolipoproteins A1
APOB: Apolipoproteins B
HCY: Homocysteine
LAZ: Length-for-age Z score
WAZ: Weight-for-age Z score
WLZ: Weight-for-length Z score
BAZ: BMI-for-age Z score
HCZ: Head-circumference-for-age Z score
