# Supplementary material for: The impact of maternal intrahepatic cholestasis during pregnancy on the growth trajectory of offspring: a population-based nested case‒control cohort study
Source: BMC Pregnancy Childbirth. 2024 Jun 7;24:413. doi: 10.1186/s12884-024-06559-z (PMC11157880; doi:10.1186/s12884-024-06559-z)
Supplement: Supplementary file 1 — Additional file 1: Supplementary Table 1. Comparison of the Neonatal Baseline Characteristics between Pregnant Patients with ICP and Patients with Normal Pregnancies. Supplementary Table 2. Diagnosis of abnormal development in offspring between the ICP group and the normal group. Supplementary Table 3. Logistic regression of abnormal development in offspring in the ICP group. Supplementary Table 4. Logistic regression of abnormal offspring development in the normal group. [file 12884_2024_6559_MOESM1_ESM.docx]

**Supplementary Table 1. Comparison of the Neonatal Baseline Characteristics between Pregnant Patients with ICP and Patients with Normal Pregnancies**

| **The baseline characteristics of neonatal** | | | |
| --- | --- | --- | --- |
| ***Between ICP and Normal Pregnancy*** | | | |
|  | ICP N=122 | Normal N=152 | P-Value*^1^* |
| **Placenta weight, g, Median (IQR)** | 510.0(107) | 541.5(110) | <0.05***** |
| **Birth weight, g, Median (IQR)** | 2830(860) | 3170(625) | <0.05***** |
| **Birth height, cm, Median (IQR)** | 48(4) | 49(3) | <0.001***** |
| **NCU, N(%)** | 68(55.7) | 82(53.9) | 0.862 |
| **NICU, N(%)** | 19(19.6) | 5(3.3) | <0.001***** |
| MSAF | 11(9.0) | 6(3.9) | 0.084 |
| Neonatal pneumonia | 15(12.3) | 7 (4.6) | 0.019***** |
| Hypoproteinemia | 2(1.6) | 0 (0.0) | 0.113 |
| Hypoglycemia | 9(7.4) | 3(1.9) | 0.029***** |
| Neonatal Anemia | 4(3.3) | 3(1.9) | 0.496 |
| PJON | 19(15.5) | 14 (9.2) | 0.108 |
| NRDS | 7(5.7) | 3(1.9) | 0.098 |
| Apnea | 1(0.8) | 0(0.0) | 0.263 |
| Respiratory Failure | 3(2.4) | 0(0.0) | 0.051 |
| SGA | 5(4.1) | 4(2.6) | 0.498 |
| Asphyxia | 2(1.6) | 1(0.6) | 0.437 |
| **Abbreviation: NCU, neonatal care unit; NICU, neonatal intensive care unit; MSAF, meconium-stained amniotic fluid; PJON, pathological jaundice of the newborn; NRDS, neonatal respiratory distress syndrome. SGA, small for gestational age.** | | | |
| ***^1^* The '*' symbol denotes P values <0.05 and are statistically significant.** | | | |

**Supplementary Table 2. Diagnosis of abnormal development in offspring between the ICP group and the normal group.**

| **The Diagnosis Of Abnormal Development in Offspring** | | | |
| --- | --- | --- | --- |
| ***Between ICP and Normal Pregnancy*** | | | |
| Characteristic | ICP N=50 | Normal N=87 | P-Value^3^ |
| Global Developmental Delay | 1(2.0) | 0(0.0) | 0.365 |
| Language Delay | 2(4.0) | 1(1.1) | 0.554 |
| Stunting^1^ | 12(24.0) | 6(6.9) | 0.004^*^ |
| Underweight^2^ | 8(16.0) | 7(8.0) | 0.166 |
| HCZ < -2 | 7(14.0) | 3(3.4) | 0.036^*^ |
| **^1^Stunting, defined as LAZ< -2;** ^2^**Underweight, defined as WAZ <-2.** | | | |
| **^3^The '*' symbol denotes P values <0.05 and are statistically significant** | | | |

**Supplementary Table 3. Logistic regression of abnormal development in offspring in the ICP group**

| Variables | OR | 95%CI Low | 95%CI Upp | P-value^1^ |
| --- | --- | --- | --- | --- |
| TBA (10-40) | 2.05 | 0.71 | 5.88 | 0.183 |
| TBA (>=40) | 5.85 | 1.55 | 22.04 | 0.009^*^ |
| LDH | 1.004 | 1.00 | 1.01 | 0.012^*^ |
| ^1^The '*' symbol denotes P values <0.05 and are statistically significant | | | | |

**Supplementary Table 4. Logistic regression of abnormal offspring development in the normal group**

| Variables | RR | 95%CI Low | 95%CI Upp | P-value |
| --- | --- | --- | --- | --- |
| LDH | 1.003 | 0.98 | 1.01 | 0.667 |
